# Supplementary material for: Chemical Constituents of Cassia abbreviata and Their Anti-HIV-1 Activity
Source: Molecules. 2021 Apr 23;26(9):2455. doi: 10.3390/molecules26092455 (PMC8122790; doi:10.3390/molecules26092455)
Supplement: Supplementary file 1 [file molecules-26-02455-s001.zip › molecules-1179204-supplementary.pdf]

Supplementary Materials

Chemical Constituents of *Cassia abbreviata* and Their Anti-HIV-1 Activity

Xian-Wen Yang [1,2], Zhi-Hui He [2], Yue Zheng [1], Ning Wang [1], Martin Mulinge [3,4], Jean-Claude Schmit [3,5], André Steinmetz [1] and Carole Seguin-Devaux [3]*

[1] Laboratory of Cellular and Molecular Oncology, Luxembourg Institute of Health, L-1445, Luxembourg, Luxembourg
[2] Key Laboratory of Marine Biogenetic Resources, Third Institute of Oceanography, Ministry of Natural Sources, 184 Daxue Road, Xiamen, Fujian 361005, China
[3] Department of Infection and Immunity, Luxembourg Institute of Health, L-4354 Esch-sur-Alzette, Luxembourg
[4] Department of Biochemistry, School of Medicine, University of Nairobi. P.O Box 30197 – 00100 Nairobi Kenya
[5] Service National of Infectious Diseases, Centre Hospitalier de Luxembourg, L-1210 Luxembourg
* Correspondence: caroledevaux@lih.lu;

Content

Figure S1. ^{1}H NMR spectrum of **1** in DMSO-*d6*.

Figure S2. ^{13}C NMR spectrum of **1** in DMSO-*d6*.

Figure S3. HSQC NMR spectrum of **1** in DMSO-*d6*.

Figure S4. HMBC NMR spectrum of **1** in DMSO-*d6*.

Figure S5. ^{1}H NMR spectrum of **1** in CD_3OD.

Figure S6. ^{13}C NMR spectrum of **1** in CD_3OD.

Figure S7. HSQC NMR spectrum of **1** in CD_3OD.

Figure S8. HMBC NMR spectrum of **1** in CD_3OD.

Figure S9. ^{1}H NMR spectrum of **2** in CD_3OD.

Figure S10. ^{13}C NMR spectrum of **2** in CD_3OD.

Figure S11. HSQC NMR spectrum of **2** in CD_3OD.

Figure S12. HMBC NMR spectrum of **2** in CD_3OD.

Figure S13. ^{1}H NMR spectrum of **3** in CD_3OD.

Figure S14. ^{13}C NMR spectrum of **3** in CD_3OD.

Figure S15. HSQC NMR spectrum of **3** in CD_3OD.

Figure S16. COSY NMR spectrum of **3** in CD_3OD.

Figure S17. HMBC NMR spectrum of **3** in CD_3OD.

Figure S18. HRESIMS spectrum of **1**.

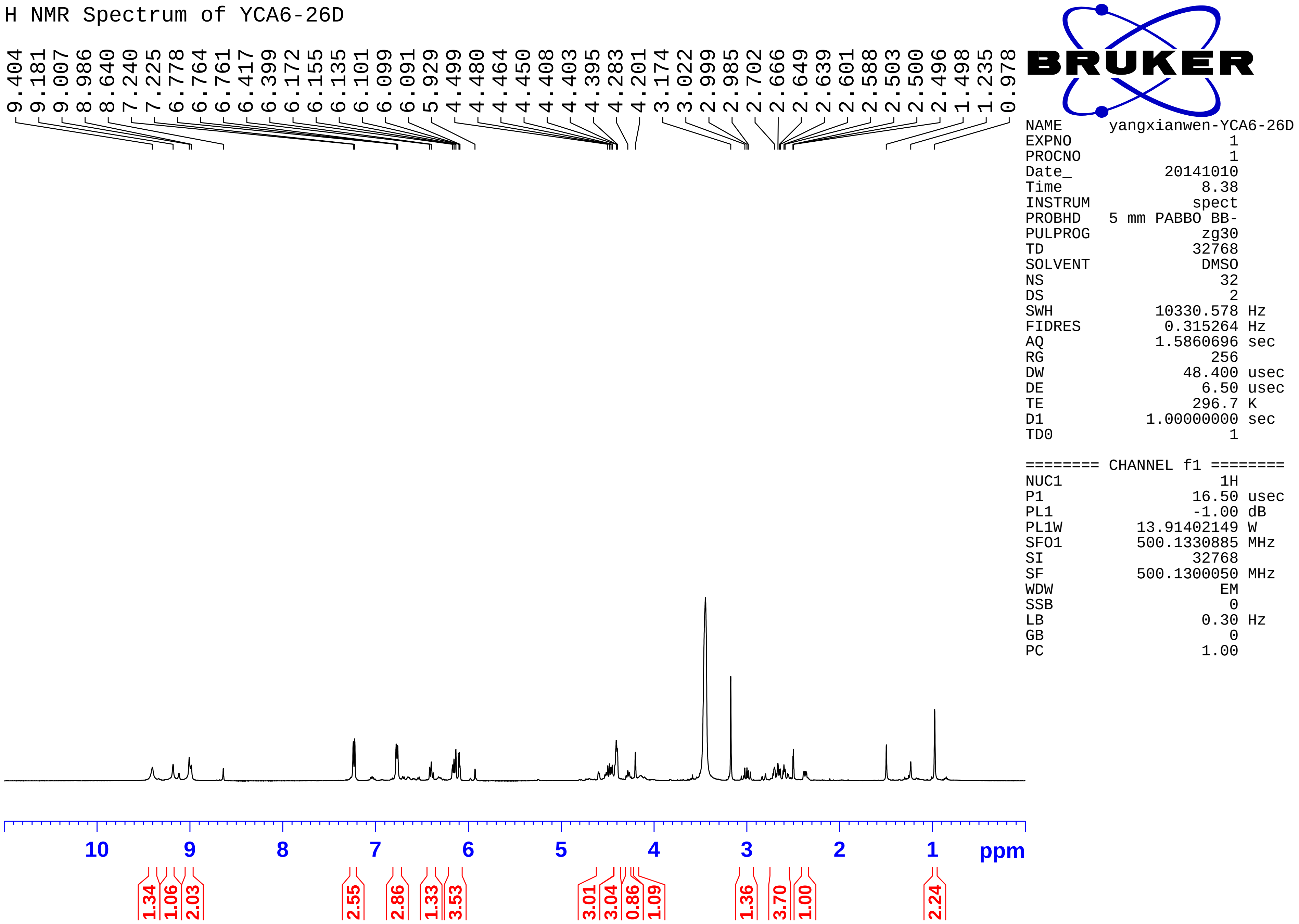

Figure S1. ^{1}H NMR spectrum of **1** in DMSO-*d6*.

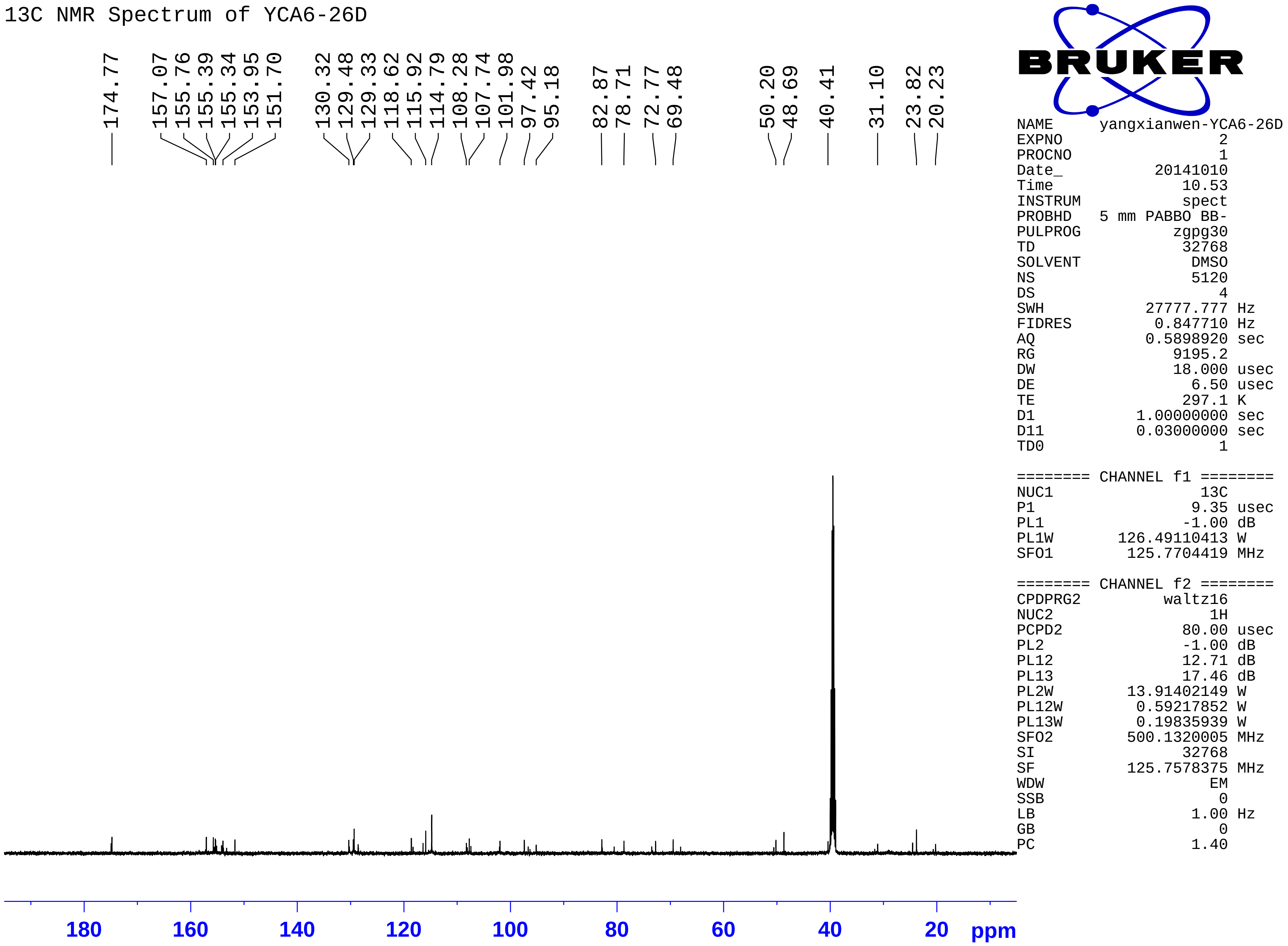

Figure S2. ^{13}C NMR spectrum of **1** in DMSO-*d6*.

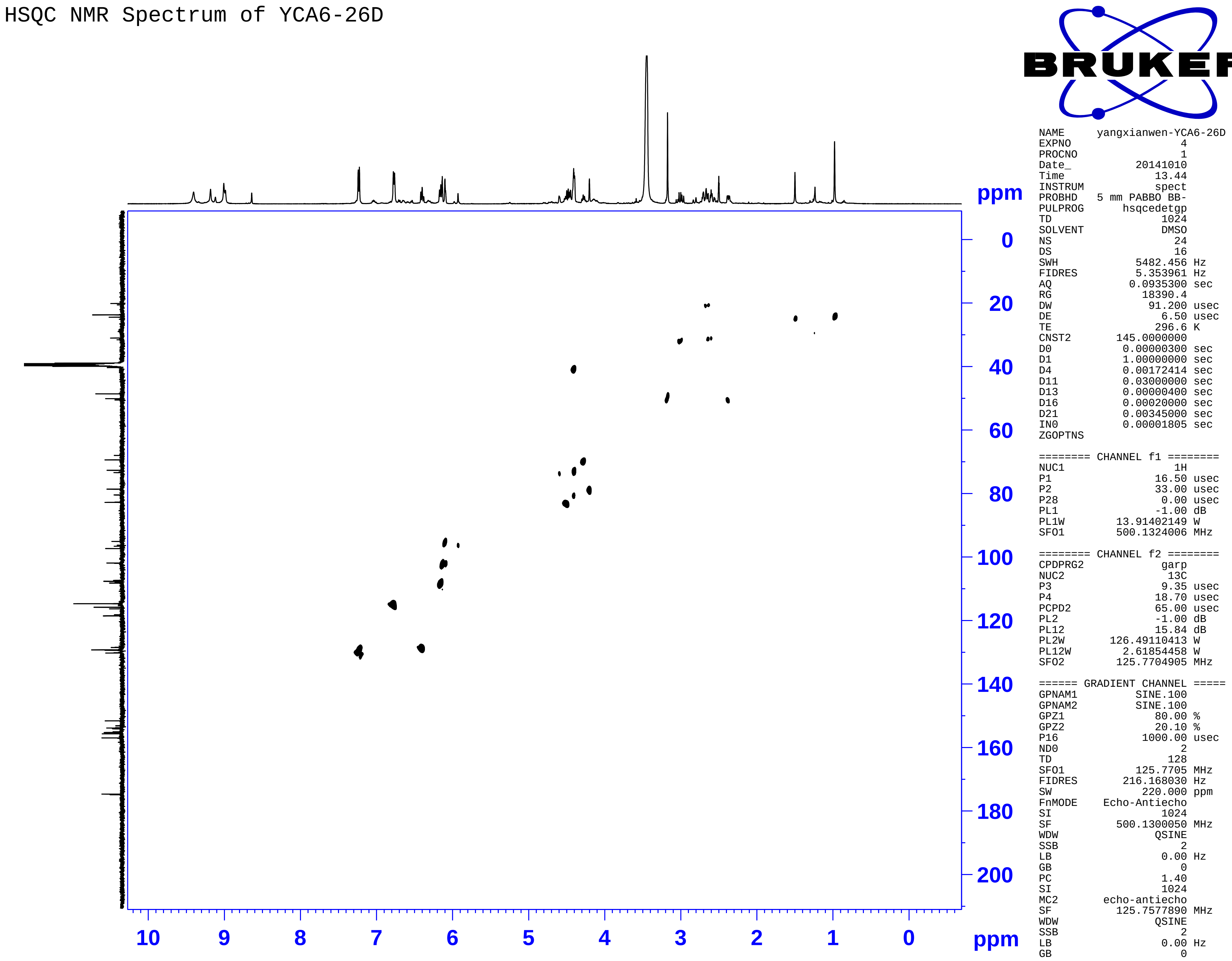

Figure S3. HSQC NMR spectrum of **1** in DMSO-*d6*.

HMBC NMR Spectrum of YCA6-26D

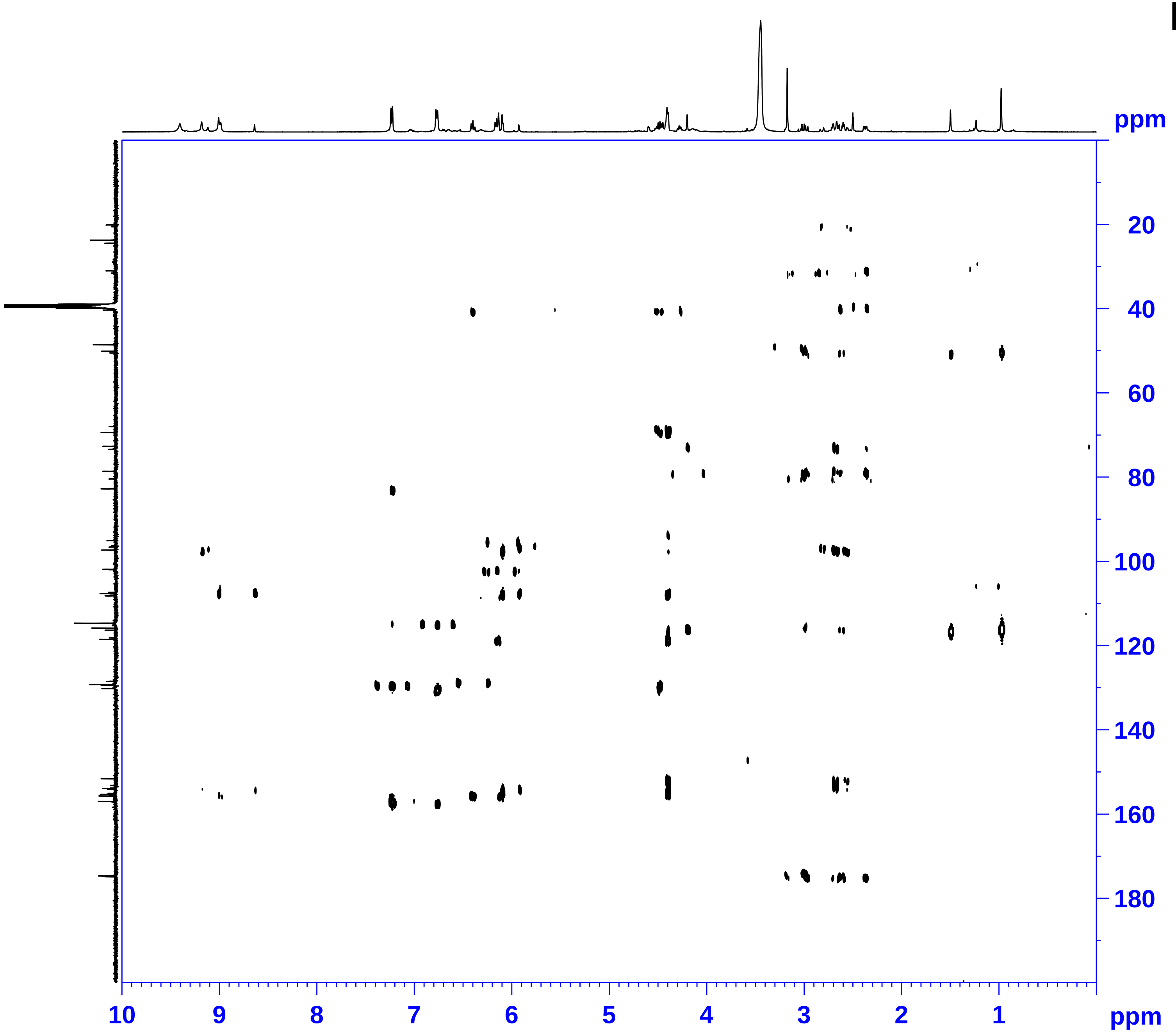


```
NAME        yangxianwen-YCA6-26D
EXPNO                  3
PROCNO                 1
Date_           20141010
Time               11.16
INSTRUM            spect
PROBHD   5 mm PABBO BB-
PULPROG       hmbcgpndqf
TD                  4096
SOLVENT             DMSO
NS                    48
DS                    16
SWH             5482.456 Hz
FIDRES          1.338490 Hz
AQ             0.3736964 sec
RG               23170.5
DW                91.200 usec
DE                  6.50 usec
TE                 296.9 K
CNST13         8.0000000
D0            0.00000300 sec
D1            1.00000000 sec
D6            0.06250000 sec
D16           0.00020000 sec
IN0           0.00001805 sec

======== CHANNEL f1 ========
NUC1                  1H
P1                 16.50 usec
P2                 33.00 usec
PL1                -1.00 dB
PL1W         13.91402149 W
SFO1         500.1324006 MHz

======== CHANNEL f2 ========
NUC2                 13C
P3                  9.35 usec
PL2                -1.00 dB
PL2W        126.49110413 W
SFO2         125.7704905 MHz

====== GRADIENT CHANNEL =====
GPNAM1          SINE.100
GPNAM2          SINE.100
GPNAM3          SINE.100
GPZ1               50.00 %
GPZ2               30.00 %
GPZ3               40.10 %
P16              1000.00 usec
ND0                    2
TD                   128
SFO1            125.7705 MHz
FIDRES        216.168030 Hz
SW               220.000 ppm
FnMODE                QF
SI                  1024
SF           500.1300050 MHz
WDW                 SINE
SSB                    0
LB                  0.00 Hz
GB                     0
PC                  1.40
SI                  1024
MC2                   QF
SF           125.7577890 MHz
WDW                 SINE
SSB                    0
LB                  0.00 Hz
GB                     0
```

Figure S4. HMBC NMR spectrum of **1** in DMSO-*d6*.

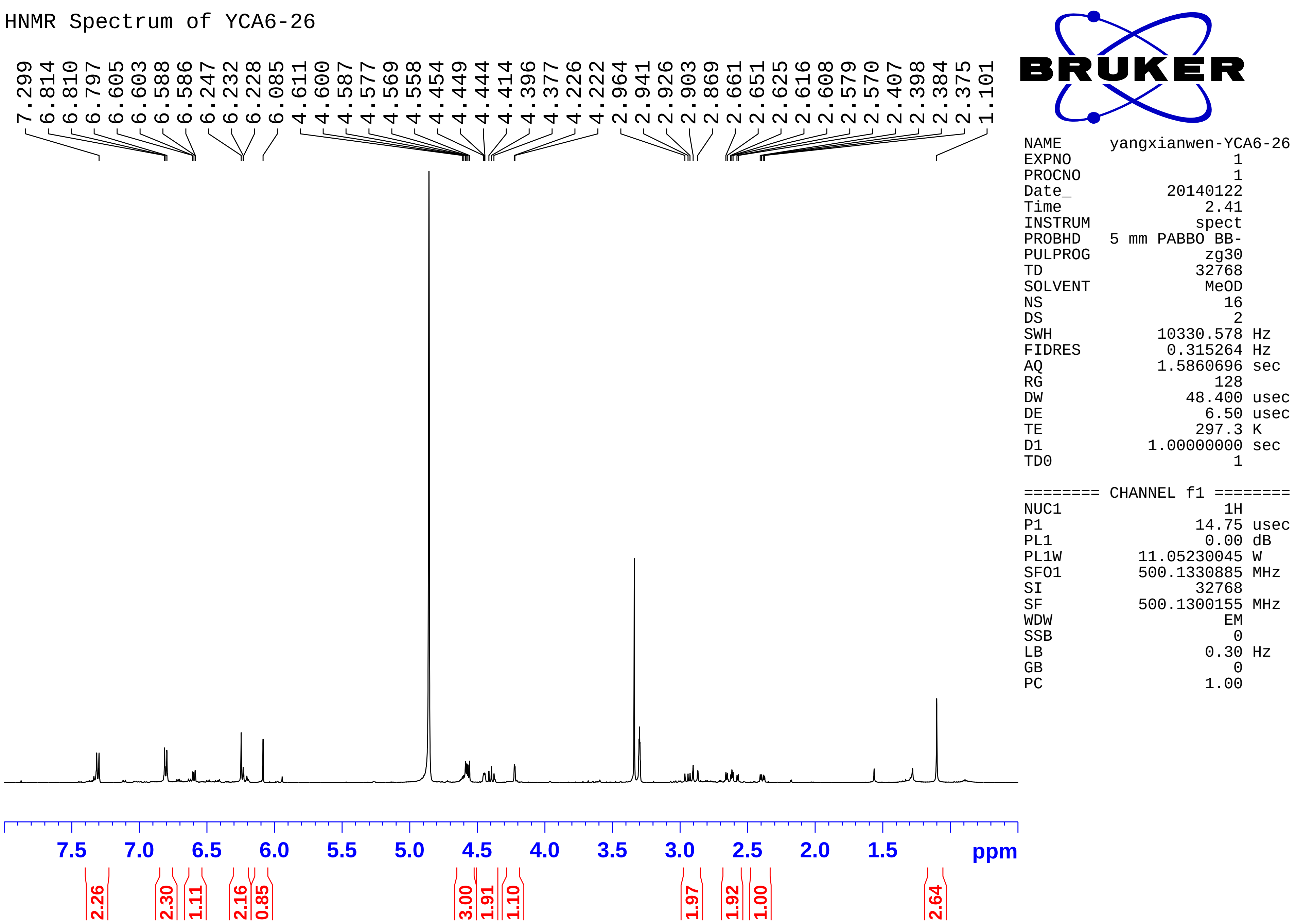

Figure S5. ^{1}H NMR spectrum of **1** in CD_3OD.

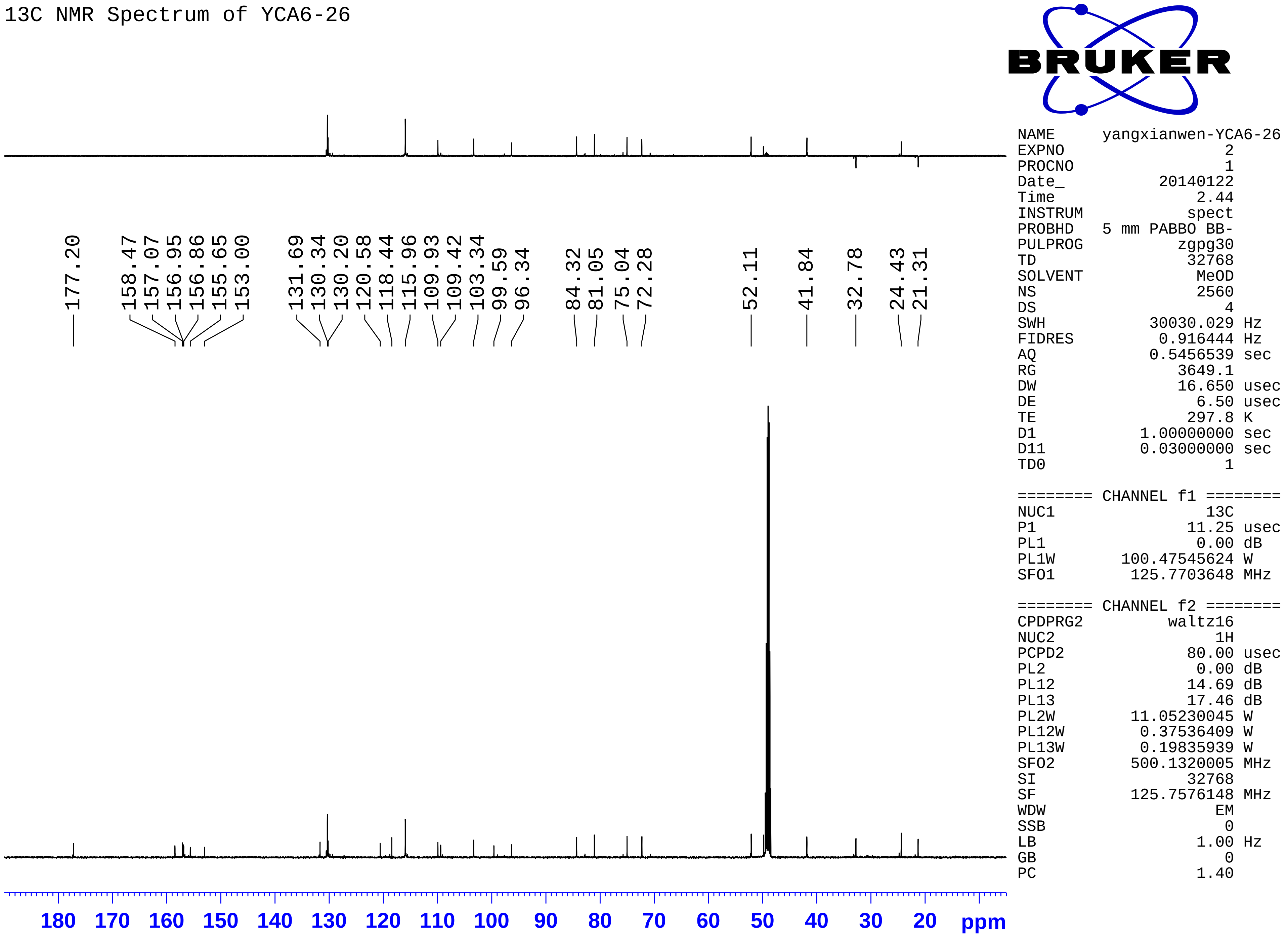

Figure S6. ^{13}C NMR spectrum of **1** in CD_3OD.

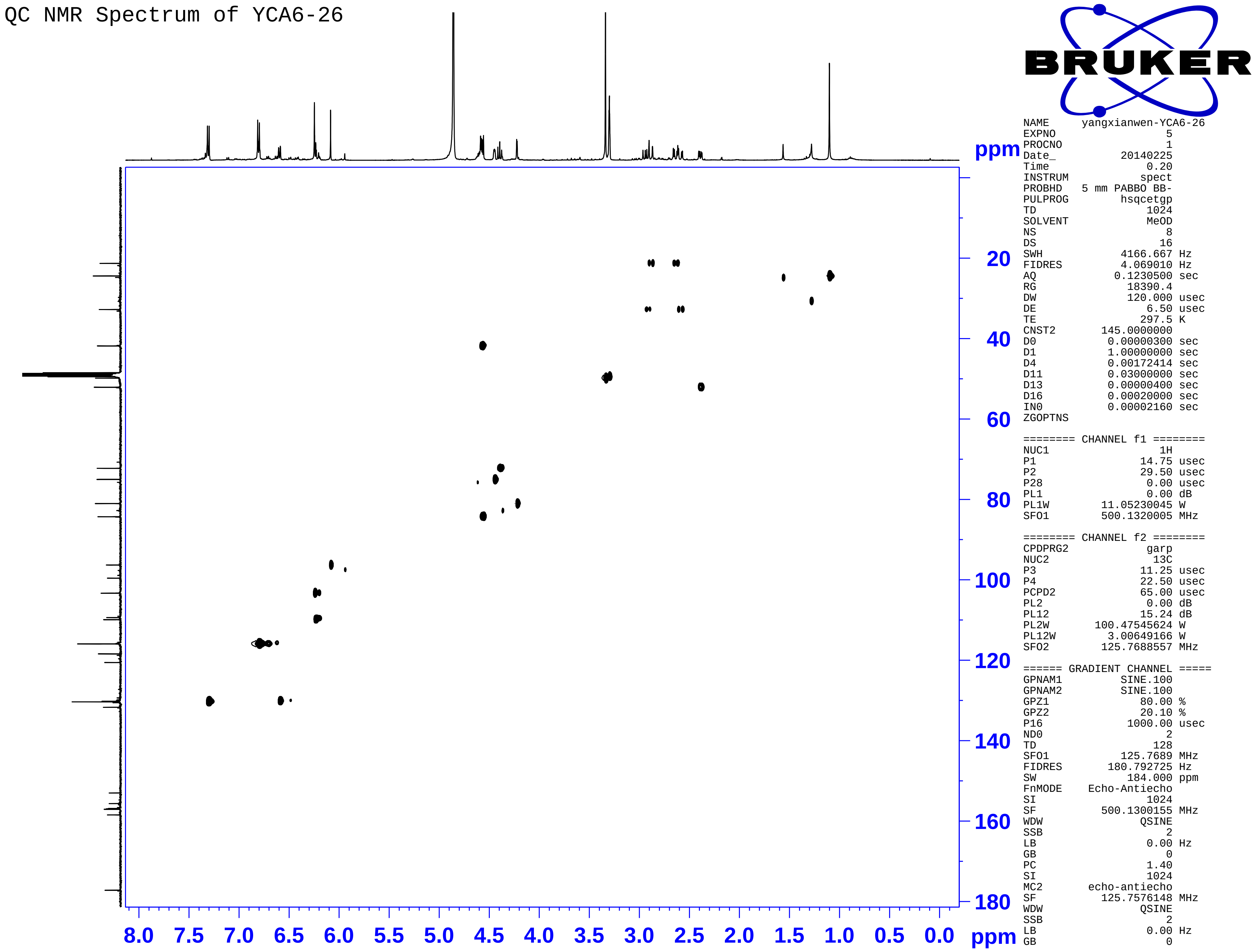

Figure S7. HSQC NMR spectrum of **1** in CD_3OD.

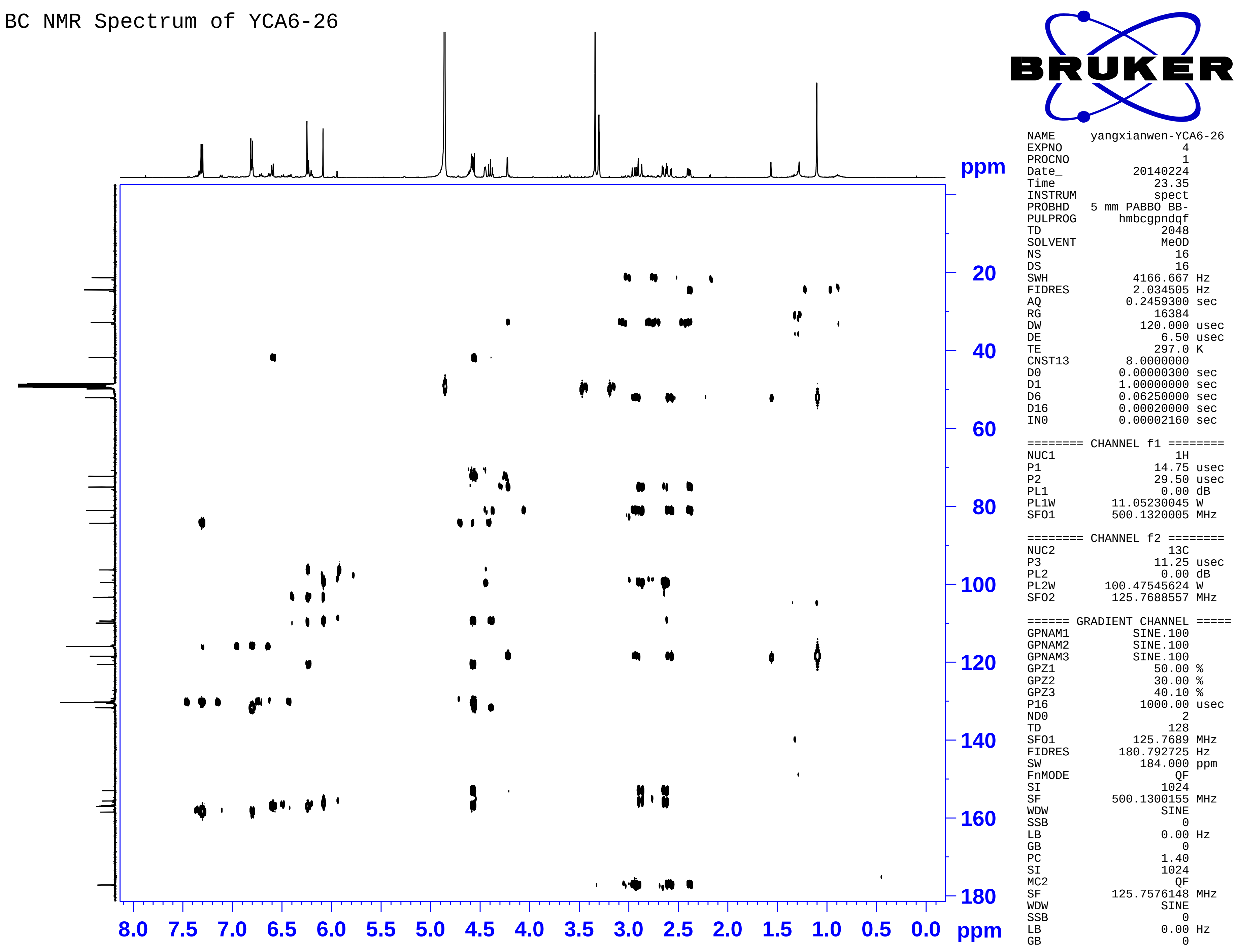

Figure S8. HMBC NMR spectrum of **1** in CD_3OD.

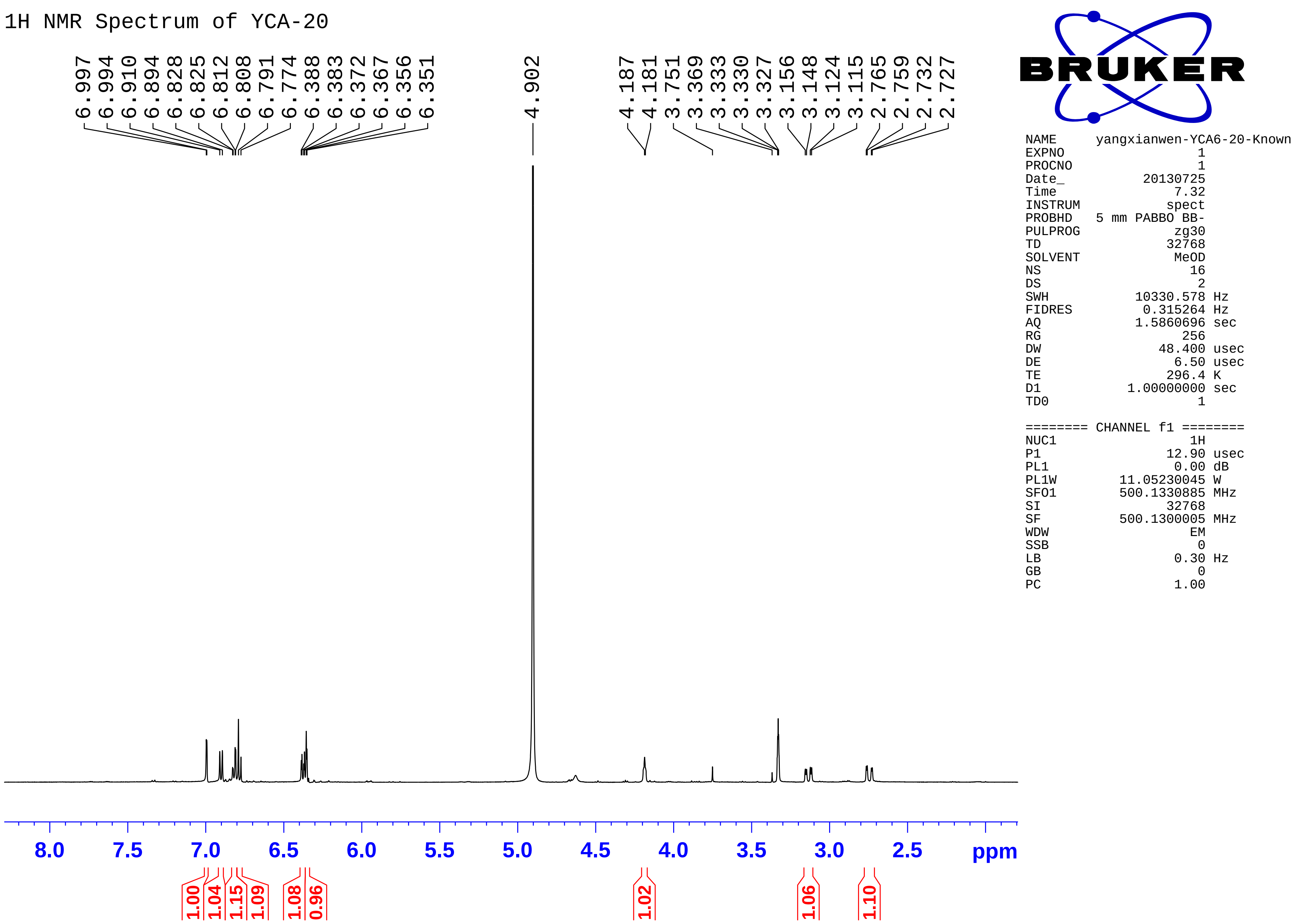

Figure S9. ^{1}H NMR spectrum of **2** in CD_3OD.

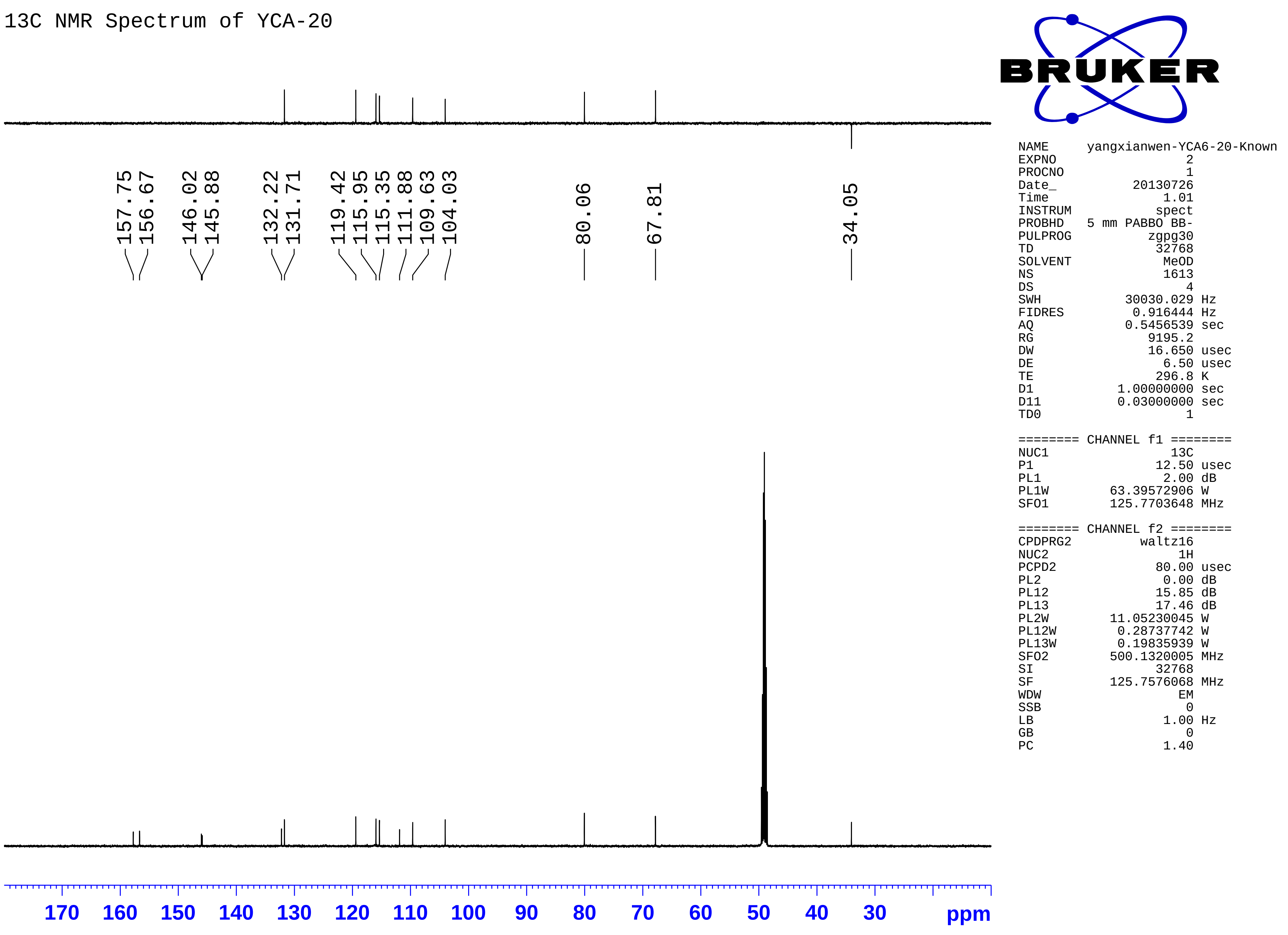

Figure S10. ^{13}C NMR spectrum of **2** in CD_3OD.

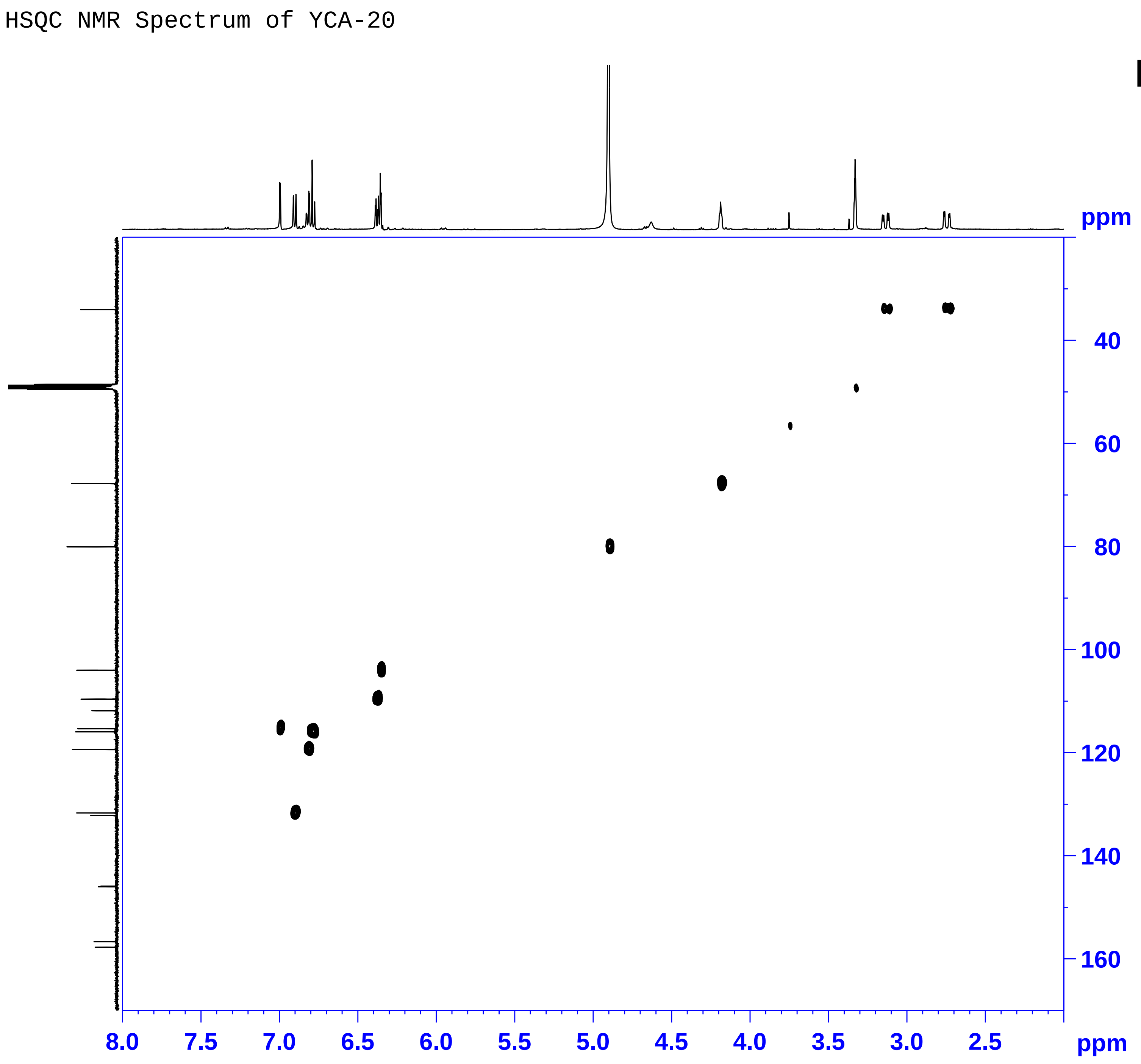


```
NAME          yangxianwen-YCA6-20-Known
EXPNO                     5
PROCNO                    1
Date_              20130727
Time                   2.02
INSTRUM               spect
PROBHD      5 mm PABBO BB-
PULPROG          hsqcedetgp
TD                     1024
SOLVENT                MeOD
NS                        8
DS                       16
SWH                5208.333 Hz
FIDRES             5.086263 Hz
AQ                0.0984500 sec
RG                  18390.4
DW                   96.000 usec
DE                     6.50 usec
TE                    296.1 K
CNST2           145.0000000
D0               0.00000300 sec
D1               1.00000000 sec
D4               0.00172414 sec
D11              0.03000000 sec
D13              0.00000400 sec
D16              0.00020000 sec
D21              0.00345000 sec
IN0              0.00001890 sec
ZGOPTNS

======== CHANNEL f1 ========
NUC1                     1H
P1                    12.90 usec
P2                    25.80 usec
P28                    0.00 usec
PL1                    0.00 dB
PL1W            11.05230045 W
SFO1            500.1323006 MHz

======== CHANNEL f2 ========
CPDPRG2                garp
NUC2                    13C
P3                    12.50 usec
P4                    25.00 usec
PCPD2                 65.00 usec
PL2                    2.00 dB
PL12                  16.32 dB
PL2W            63.39572906 W
PL12W            2.34455276 W
SFO2            125.7697360 MHz

====== GRADIENT CHANNEL =====
GPNAM1             SINE.100
GPNAM2             SINE.100
GPZ1                  80.00 %
GPZ2                  20.10 %
P16                 1000.00 usec
ND0                       2
TD                      128
SFO1               125.7697 MHz
FIDRES           206.635742 Hz
SW                  210.300 ppm
FnMODE         Echo-Antiecho
SI                     1024
SF              500.1300005 MHz
WDW                   QSINE
SSB                       2
LB                     0.00 Hz
GB                        0
PC                     1.40
SI                     1024
MC2            echo-antiecho
SF              125.7576068 MHz
WDW                   QSINE
SSB                       2
LB                     0.00 Hz
GB                        0
```

Figure S11. HSQC NMR spectrum of **2** in CD_3OD.

HMBC NMR Spectrum of YCA-20

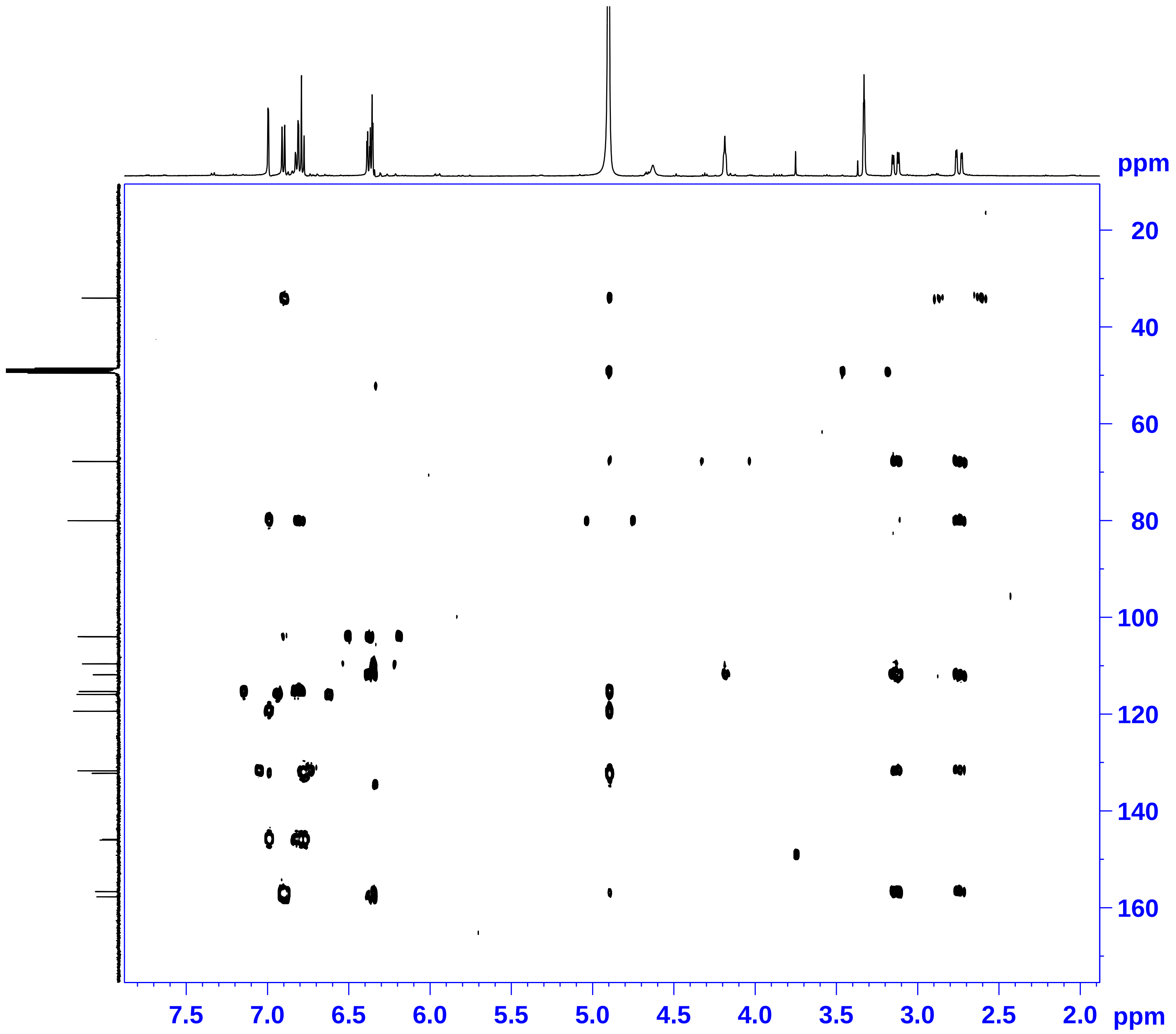


```
NAME       yangxianwen-YCA6-20-Known
EXPNO                    4
PROCNO                   1
Date_             20130727
Time                  1.12
INSTRUM              spect
PROBHD   5 mm PABBO BB-
PULPROG         hmbcgpndqf
TD                    4096
SOLVENT               MeOD
NS                      16
DS                      16
SWH               5208.333 Hz
FIDRES            1.271566 Hz
AQ               0.3933620 sec
RG                   26008
DW                  96.000 usec
DE                    6.50 usec
TE                   296.1 K
CNST13           8.0000000
D0              0.00000300 sec
D1              1.00000000 sec
D6              0.06250000 sec
D16             0.00020000 sec
IN0             0.00001890 sec

======== CHANNEL f1 ========
NUC1                    1H
P1                   12.90 usec
P2                   25.80 usec
PL1                   0.00 dB
PL1W           11.05230045 W
SFO1           500.1323006 MHz

======== CHANNEL f2 ========
NUC2                   13C
P3                   12.50 usec
PL2                   2.00 dB
PL2W           63.39572906 W
SFO2           125.7697360 MHz

====== GRADIENT CHANNEL =====
GPNAM1           SINE.100
GPNAM2           SINE.100
GPNAM3           SINE.100
GPZ1                50.00 %
GPZ2                30.00 %
GPZ3                40.10 %
P16               1000.00 usec
ND0                     2
TD                    128
SFO1             125.7697 MHz
FIDRES         206.635742 Hz
SW                210.300 ppm
FnMODE                 QF
SI                   1024
SF            500.1300005 MHz
WDW                  SINE
SSB                     0
LB                   0.00 Hz
GB                      0
PC                   1.40
SI                   1024
MC2                    QF
SF            125.7576068 MHz
WDW                  SINE
SSB                     0
LB                   0.00 Hz
GB                      0
```

Figure S12. HMBC NMR spectrum of **2** in CD_3OD.

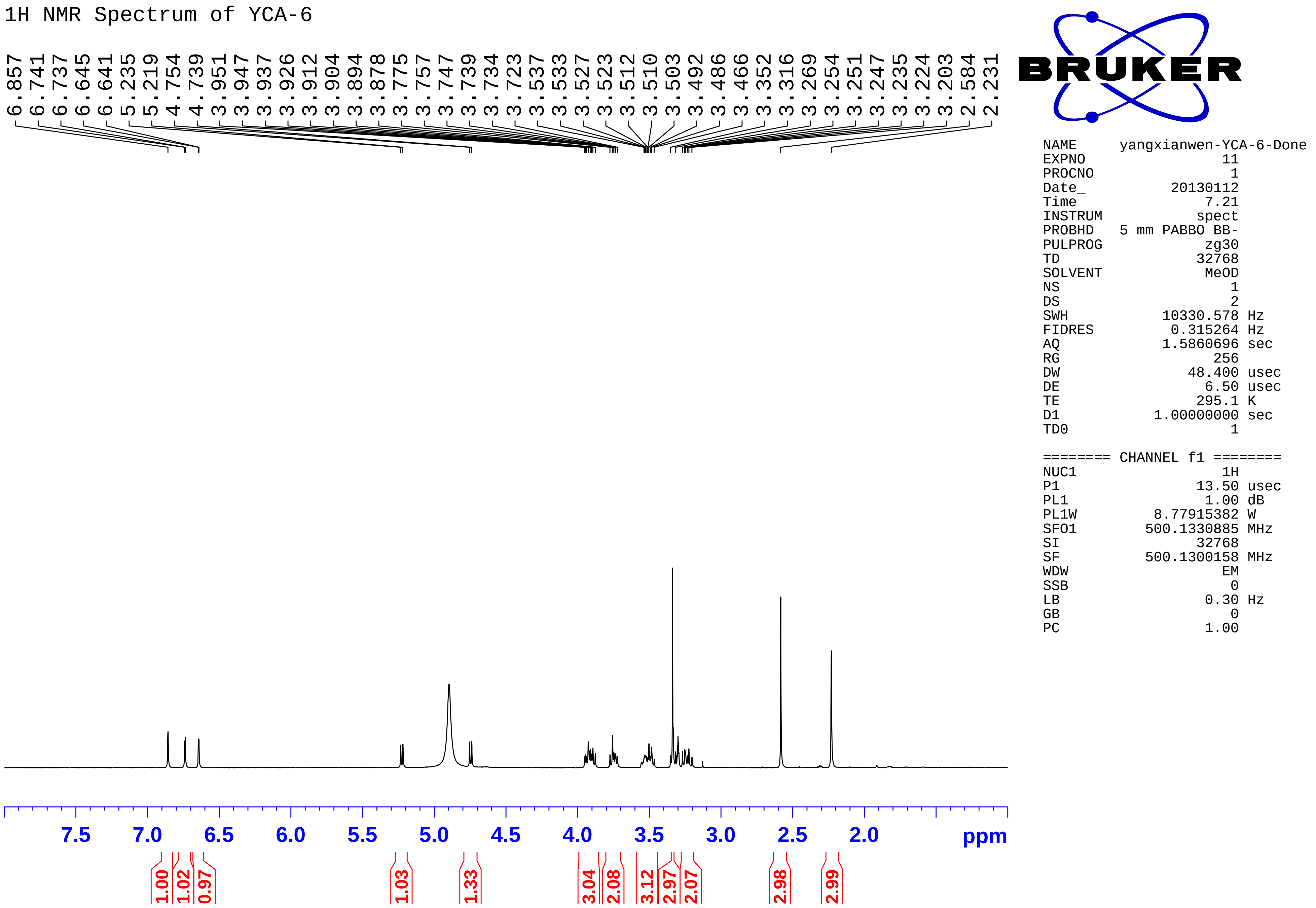

Figure S13. ^{1}H NMR spectrum of **3** in CD_3OD.

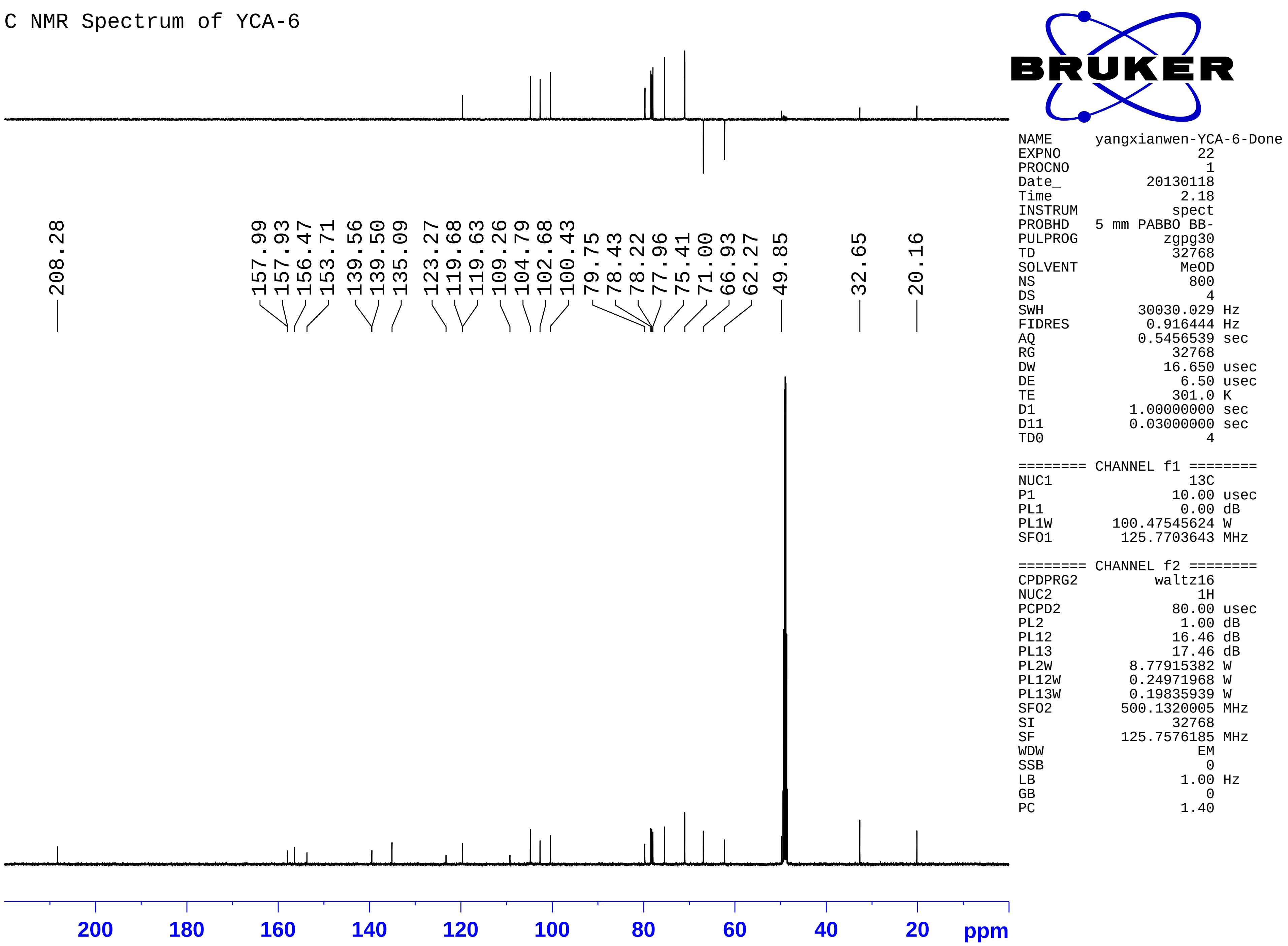

Figure S14. ^{13}C NMR spectrum of **3** in CD_3OD.

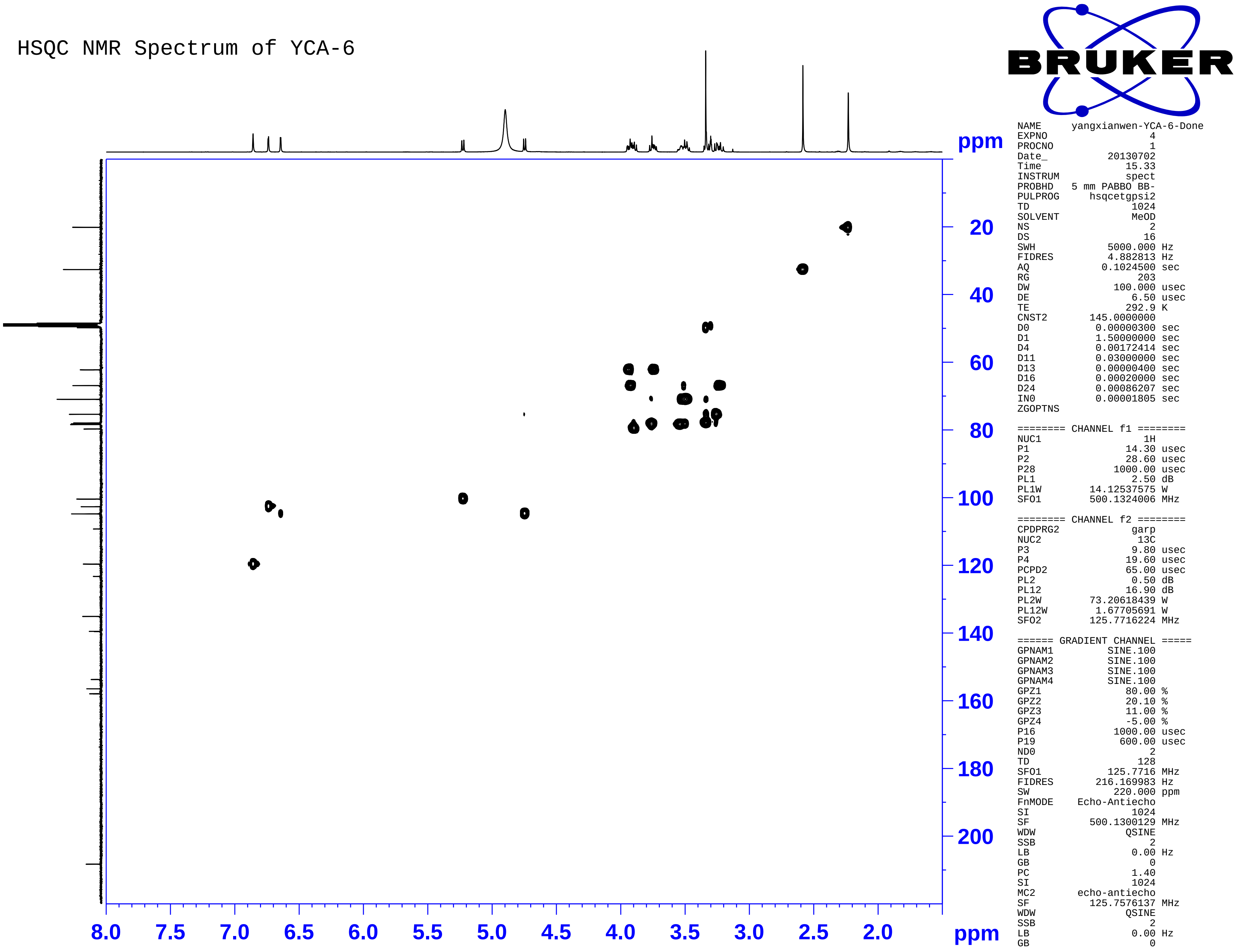

Figure S15. HSQC NMR spectrum of **3** in CD_3OD.

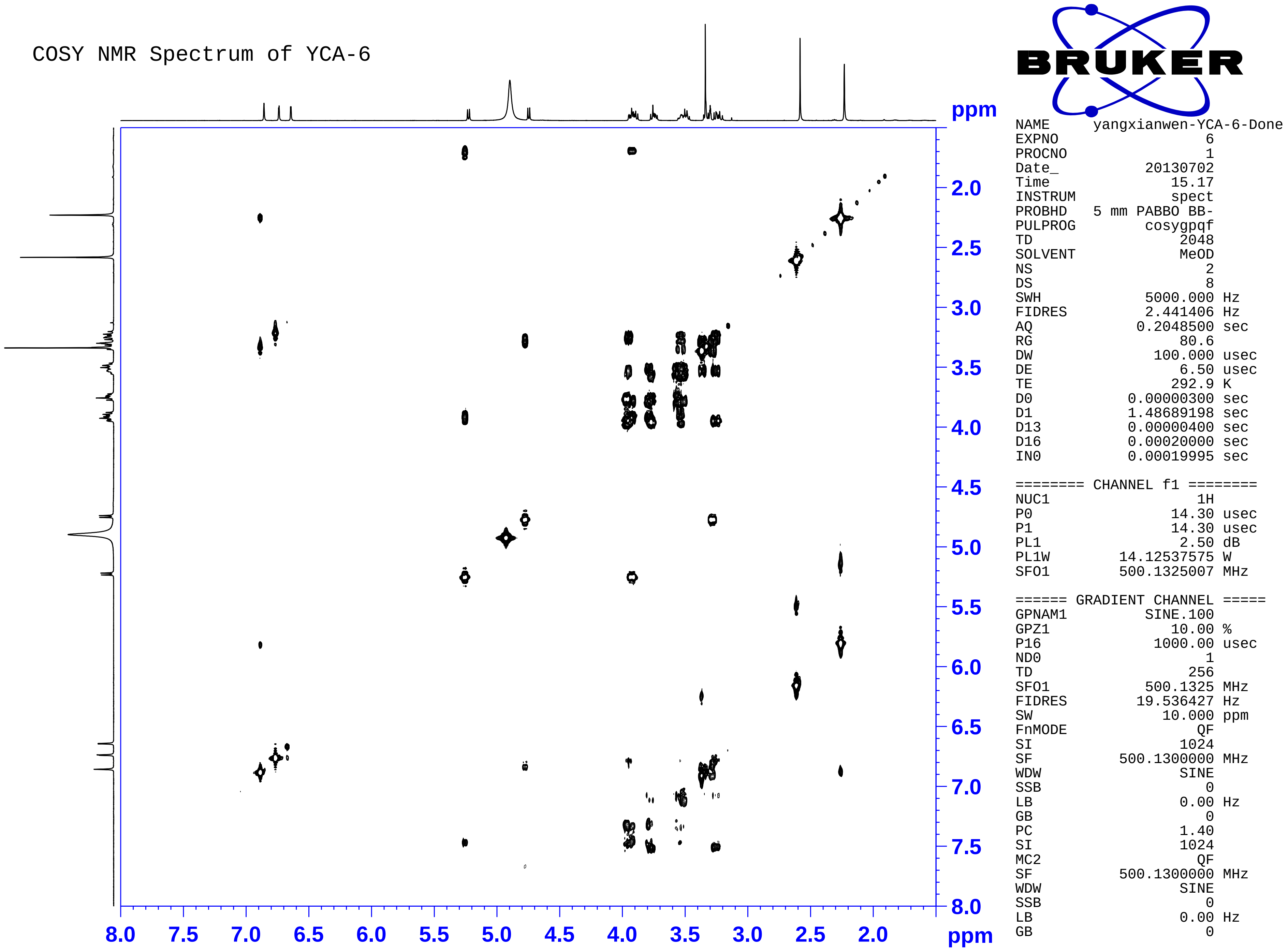

Figure S16. COSY NMR spectrum of **3** in CD_3OD.

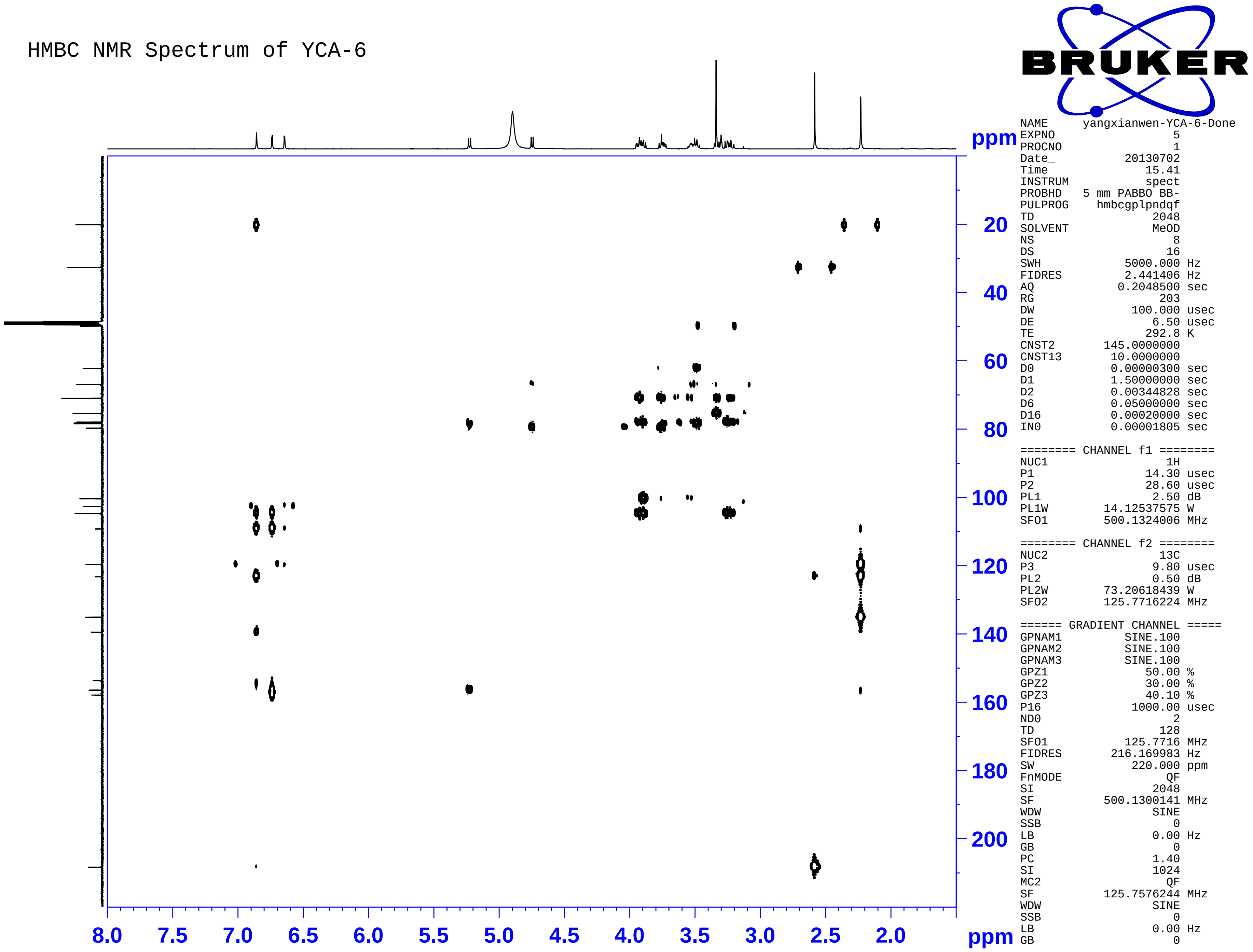

Figure S17. HMBC NMR spectrum of **3** in CD_3OD.

Mass Spectrum SmartFormula Report

Analysis Info

Analysis Name	D:\Data\MS\data\201402\yangxianwen_6-26_pos.d	Acquisition Date	2/21/2014 2:50:22 PM
Method	POS_100-2000_Dirrect Infusion.m	Operator	SCSIO
Sample Name	SCSIO	Instrument / Ser#	maXis 29
Comment			

Acquisition Parameter

Source Type	ESI	Ion Polarity	Positive	Set Nebulizer	0.3 Bar
Focus	Not active	Set Capillary	4500 V	Set Dry Heater	180 °C
Scan Begin	100 m/z	Set End Plate Offset	-500 V	Set Dry Gas	4.0 l/min
Scan End	2000 m/z	Set Collision Cell RF	2000.0 Vpp	Set Divert Valve	Waste

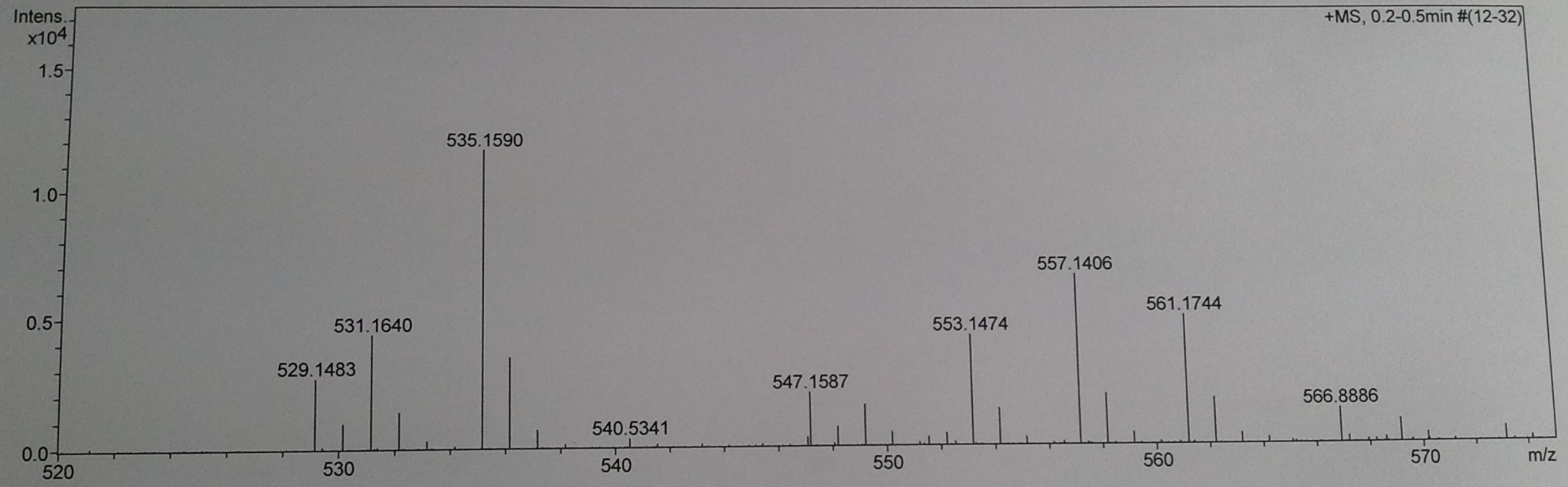

Meas. m/z	#	Formula	Score	m/z	err [mDa]	err [ppm]	mSigma	rdb	e⁻ Conf	N-Rule
535.1590	1	C 25 H 23 N 6 O 8	50.75	535.1572	-1.8	-3.4	4.6	17.5	even	ok
	2	C 29 H 27 O 10	81.21	535.1599	0.9	1.7	10.7	16.5	even	ok
	3	C 26 H 19 N 10 O 4	100.00	535.1585	-0.5	-0.9	11.2	22.5	even	ok
	4	C 30 H 23 N 4 O 6	26.84	535.1612	2.2	4.2	20.7	21.5	even	ok
557.1406	1	C 25 H 22 N 6 Na O 8	58.17	557.1391	-1.5	-2.6	6.2	17.5	even	ok
	2	C 29 H 26 Na O 10	60.66	557.1418	1.2	2.2	11.9	16.5	even	ok
	3	C 26 H 18 N 10 Na O 4	100.00	557.1405	-0.1	-0.2	15.3	22.5	even	ok
	4	C 30 H 22 N 4 Na O 6	17.95	557.1432	2.6	4.6	22.7	21.5	even	ok

Bruker Compass DataAnalysis 4.0 printed: 2/21/2014 3:02:05 PM Page 1 of 1

Figure S18. HRESIMS spectrum of 1.
